# 2.N. Workshop: Improving perinatal health and reducing inequality: the value of European population comparisons

**DOI:** 10.1093/eurpub/ckac129.108

**Published:** 2022-10-25

**Authors:** Organised by: Euro-Peristat Network, Chair persons: Guenther Heller (Germany), Mika Gissler (Finland)

## Abstract

In Europe, maternal and child mortality and morbidity during pregnancy and childbirth have declined markedly since the turn of the 20th century, but recent data suggest this trend may be slowing or reversing. Improvements in perinatal health were due primarily to increases in overall standards of living and clinical advances in obstetrics and neonatology which successfully increased maternal and newborn survival when pregnancy complications arose. However, fewer gains have been made in prevention and, in many countries, preterm birth and low birthweight rates have increased. To further improve perinatal health, a population-level approach focusing on prevention and appropriate use of clinical interventions is required. Prevention strategies include reducing risk factors (e.g., smoking and obesity) and ensuring universal access to high-quality health care due to the key roles played by early antenatal care, regular antenatal appointments and referral pathways in the timely identification and management of pregnancy complications. A population approach is particularly essential because both the burden of disease and the dangers of poor care organization fall disproportionately on socially disadvantaged women and babies, contributing to lifelong health inequalities. A final challenge is to avoid over-medicalising pregnancy and childbirth for the large majority of women with uncomplicated pregnancies. Medical technology has contributed greatly to the decline in maternal and infant mortality and morbidity, but clinical intervention can carry risks and must be used appropriately to optimise health outcomes. Despite commonalties such as universal access to health care and access to scientific knowledge, perinatal health outcomes and approaches to maternity care differ greatly between European countries. In this context, comparisons between different European models can be powerful tools for identifying population risk factors, assessing care practices, setting targets for population policies and for understanding their strengths and weaknesses to provide insight into the efficacy of health and medical policies. Currently, European comparisons are limited by the availability, timeliness and quality of population data on maternal and newborn health. This workshop reports on a new protocol implemented by the Euro-Peristat network to provide comparable perinatal indicators from countries across Europe. Based on select core indicators collected using a common protocol, we provide a proof of concept study for a future health information system and report up-to-date data on perinatal outcomes. The four presentations in this workshop present this protocol, describe most recent trends and disparities between countries, explore social inequalities in perinatal outcomes across Europe and raise questions about approaches which can achieve low mortality and morbidity while keeping intervention rates low.

**Key messages:**

• A federated analytical approach is an efficient and feasible way to collect timely, high-quality and comparable population data on perinatal health in Europe.

• Marked disparities in perinatal health remain between and within European countries. Our results demonstrate a need for targeted policies in many countries and offer data to inform these initiatives.

